# The interactive effects of work–family conflict and coping styles on occupational fatigue among endoscopy nurses: a cross-sectional study

**DOI:** 10.3389/fpubh.2025.1591088

**Published:** 2025-08-06

**Authors:** Zhi Zeng, Chunyan Fu, Sumei Zhou, Guiqiong Xie, Yazhi He, Meng Liu, Chao Liu

**Affiliations:** ^1^Department of Gastroenterology, Deyang People’s Hospital, Deyang, Sichuan, China; ^2^Pediatric Ward 2 (Children’s Blood/Cancer Ward), Sichuan Provincial People’s Hospital, Chengdu, China; ^3^Department of Neurosurgery, Deyang People’s Hospital, Deyang, Sichuan, China

**Keywords:** endoscopy nurses, occupational fatigue, work–family conflict, coping style, JD-R model, interactive effects

## Abstract

**Background:**

Although work–family conflict and coping styles have been identified as factors influencing nurses’ occupational fatigue, the interactive mechanism between these variables remains unclear among endoscopy nurses. This study, guided by the Job Demands-Resources (JD-R) Model, aims to investigate the interactive effects of work–family conflict and coping styles on occupational fatigue among endoscopy nurses.

**Methods:**

A cross-sectional study was conducted from July 1 to July 31, 2024, involving 320 endoscopy nurses from 23 medical institutions across China. Standardized scales, including the Work–Family Conflict (WFC) scale, the Simplified Coping Style Questionnaire (SCSQ), and the Fatigue Assessment Instrument (FAI), were used to measure work–family conflict, coping styles, and occupational fatigue, respectively. Logistic regression models were applied to analyze the associations between variables, and additive interaction indices (RERI, API, SI) were used to test interaction effects.

**Results:**

High-intensity work–family conflict (OR = 1.214, 95% CI: 1.143–1.289) and negative coping (OR = 1.209, 95% CI: 1.137–1.285) significantly increased the risk of occupational fatigue, whereas positive coping could reduce the risk of occupational fatigue (OR = 0.800, 95% CI: 0.755–0.848). Interaction analysis indicated that there was a synergistic effect between work–family conflict and negative coping (SI = 1.293, 95% CI: 1.064–1.489), while an antagonistic effect was observed between work–family conflict and positive coping (SI = 0.700, 95% CI: 0.205–1.990).

**Conclusion:**

Work–family conflict and negative coping jointly exacerbate occupational fatigue among endoscopy nurses through a synergistic interaction, while positive coping may help mitigate these adverse effects. Interventions aimed at optimizing work resource allocation, enhancing adaptive coping training, and improving work-family boundary management are recommended to promote occupational health in this population.

## 1 Introduction

Nursing staff, as vital members of the medical team, directly impact the quality of medical care and patient safety through their occupational health and work status. Occupational fatigue, a core issue in occupational health, is increasingly threatening the physical and mental well—being of nurses (1). Data shows that 84.96% of nurses experience varying degrees of occupational fatigue, significantly higher than that of other medical professional groups (2). This condition not only impairs work efficiency and job satisfaction but also increases the likelihood of medical errors and adverse events, posing serious risks to patient safety (3).

Endoscopy is a widely used diagnostic and therapeutic technique in clinical practice and plays an indispensable role in the diagnosis and treatment of diseases. Endoscopy nurses are directly involved in the entire process, including preoperative patient preparation, intraoperative assistance, postoperative care, and equipment maintenance (4, 5). According to the Job Demands-Resources (JD-R) model (6), when employees consistently expend physical, psychological, and social resources without adequate support, they are more likely to develop occupational fatigue. Due to factors such as heavy workloads, high infection risk, and frequent exposure to hazardous environments and equipment, endoscopy nurses face cumulative job demands that significantly elevate their risk of occupational fatigue (7). Therefore, identifying the key contributors to fatigue in this specific population and implementing effective interventions is essential to maintaining nurses’ occupational health and ensuring patient safety.

Work–family conflict is a prevalent issue among today’s workforce and is particularly pronounced among endoscopy nurses due to the unique demands of their role (8, 9). Their irregular work schedules, high-intensity tasks, and frequent on-call duties create substantial challenges in balancing professional and family responsibilities (9). Prior research has shown (10, 11) that work–family conflict significantly contributes to occupational fatigue among nurses, often resulting in emotional exhaustion, depersonalization, and diminished professional accomplishment. This conflict not only affects the quality of family life but may also exacerbate the psychological burden at work, resulting in increased psychological stress and emotional fluctuations, which in turn affect their work performance and professional status.

Coping styles play a crucial role in managing occupational fatigue, particularly under dual pressure from work and personal life (12). According to the JD-R model (13, 14), the psychological and social resources available in the work environment can help individuals respond more effectively to job demands. As an important psychological resource, coping styles influences how nurses perceive and respond to stress. Studies have indicated that adopting coping styles such as self-emotion regulation and seeking social support can enhance psychological resilience, reduce stress, and alleviate occupational fatigue (15, 16). In contrast, negative coping styles such as self-denial and procrastination can magnify stress, increase the physical and mental burden, and exacerbate occupational fatigue (17, 18). Additionally, positive coping styles can enhance job satisfaction, promote professional well-being, and help nurses maintain a healthy balance between work and family life, ultimately improving patient care outcomes (19). Therefore, cultivating positive coping styles is of great importance for nurses’ occupational health.

While numerous studies have examined the independent effects of work–family conflict and coping styles on occupational fatigue among clinical nurses, research specifically targeting endoscopy nurses remains limited. Iven the distinct nature of endoscopy nursing, findings from general nursing populations may not be directly applicable. The JD-R model highlights the dynamic interaction between job demands and job resources. In this framework, work–family conflict functions as a job demand, whereas coping style represents a job resource (9, 16, 20). Studying these factors in isolation may overlook the synergistic or buffering effects that emerge from their interaction, thus providing an incomplete understanding of fatigue development.

Therefore, grounded in the JD-R model, this study employed a cross-sectional design to explore the interactive effects of work–family conflict and coping styles on occupational fatigue among endoscopy nurses. The findings are intended to reveal potential mechanisms underlying occupational fatigue in this population and to provide a scientific basis for intervention strategies. These results may inform nursing administrators in optimizing work allocation, delivering tailored psychological support and coping training, and implementing targeted interventions to enhance the occupational health of endoscopy nurses.

## 2 Materials and methods

### 2.1 Participants

This study adopted a cross-sectional research design. Data were collected via “Wenjuanxing”, an online survey platform, from July 1st to July 31st, 2024. The study participants were endoscopy nurses from 23 medical institutions across 14 provincial-level administrative regions in China.

The inclusion criteria were: (1) having engaged in endoscopy diagnosis and treatment nursing work for at least 2 years; and (2) clinical practice for more than 80% of standard working hours over the past 6 months.

The exclusion criteria were: (1) job transfer or major changes in work processes within the past 3 months; (2) current administrative or teaching responsibilities; (3) chronic health conditions such as persistent pain or sleep disorders; and (4) a history of psychiatric medication use or psychotherapy.

Based on Kendall’s sample size estimation method (21), the required sample size was calculated as 5–10 times the number of variables. Accounting for a 20% rate of invalid responses, the estimated sample size ranged from 113 to 225. A total of 320 valid responses were ultimately included.

This study was reviewed and approved by the Ethics Committee of Deyang people’s Hospital (No. 2023-04-083-K01). All participants provided informed consent and voluntarily participated in the study.

### 2.2 Research tools

#### 2.2.1 General demographic information

This section was self-designed by the research team based on literature review and expert discussions. It includes 10 variables such as gender, age, marital status, educational level, professional title, years of work experience, daily working hours, weekly working days, fertility status, and monthly income.

#### 2.2.2 Work–Family Conflict Scale

In this study, we adopted the Work Family Conflict Scale (WFC) developed by Carlson et al. (22) and translated and culturally adapted by Bai Jing (23). This scale includes two dimensions: work-to-family conflict and family-to-work conflict, comprising a total of 18 items (e.g., “I spend too much time at work, which makes me have insufficient time to participate in family activities”). Each item is rated on a five-point Likert scale ranging from 1 (“strongly disagree”) to 5 (“strongly agree”). The total score ranges from 18 to 90, with higher scores indicating more severe work–family conflict. The scale has demonstrated good reliability and validity in Chinese nursing populations, with a Cronbach’s *α* coefficient of 0.868 (24).

#### 2.2.3 Simple Coping Style Scale

In this study, the Simple Coping Style Questionnaire (SCSQ) developed by Folkman et al. (25) and translated and culturally adapted by Xie Yaning (26) was used for measurement. This scale includes two dimensions: positive coping and negative coping, comprising a total of 20 items (e.g., “Relieve stress through work, study, or other activities”). A four-point Likert scale was used for scoring, where 0 to 3 represent “never use” to “often use” respectively. The total score range is 0–36 for positive coping and 0–24 points for negative coping, with higher scores indicating more frequent use of the corresponding coping strategy. The scale has demonstrated good psychometric properties in the Chinese population. Previous studies reported a Cronbach’s *α* coefficient of 0.750 among Chinese nurses, indicating good internal consistency (27).

#### 2.2.4 Occupational Fatigue Scale

In this study, the Fatigue Assessment Instrument (FAI) developed by Schwartz et al. (28) and translated into Chinese by Wang Tianfang’s team (29) was used. This scale includes four dimensions: (1) fatigue severity; (2) environmental specificity; (3) consequences of fatigue; and (4) response to rest and sleep, with a total of 29 items (e.g., “Fatigue affects my work, family, or life”). A 7-point Likert scale was used for scoring, where 1 to 7 represent “strongly disagree” to “strongly agree” respectively. Scores for each dimension are calculated as the average of the items within that dimension. The total score ranges from 4 to 28, with higher scores indicating a greater severity of fatigue. Previous studies have shown that the scale is reliable when applied to Chinese endoscopy nurses, with a Cronbach’s *α* coefficient was 0.851 (9).

### 2.3 Data collection

In this study, “Wenjuanxing” was used for online data collection. First, the research team established a collaborative relationship with the nursing managers of the endoscopy centers in each participating unit. Through online meetings, the research team provided a detailed explanation of the research protocol (including the research purpose, implementation process, and data security protection measures) and obtained authorization from the departments. Subsequently, the survey link was pushed to the target population via WeChat groups, and the following technical control parameters were set to ensure data quality: (1) mandatory response to prevent missing data. (2) Dual identification technology using IP addresses and device IDs was adopted to prevent repeated responses. (3) Response time threshold (10–30 min) to filter invalid responses.

In this study, a three-level quality control system was established: (1) automatic screening for patterned responses. (2) Manual review of logical consistency across key variables. (3) Independent review of flagged responses by two researchers, with follow-up where necessary. Of the 325 questionnaires collected in this study, five abnormal questionnaires were excluded (including 2 with regular response patterns and 3 with logical contradictions). Finally, 320 valid questionnaires were obtained, with a valid response rate of 98.46%.

### 2.4 Statistical analysis

All analyses were conducted using Stata 16.0. Step 1, The chi-square test were used to examine group differences. Step 2, Logistic regression model were employed to explore associations between work–family conflict, coping styles, and occupational fatigue. Step 3, The relative excess risk due to interaction (RERI), attributable proportion of interaction (API), and synergy index (SI).

Generally, if there is an additive interaction between two factors, the 95% confidence intervals (CIs) of RERI and API do not contain 0, and the 95% CI of SI does not contain 1. When the SI is greater than 1, it indicates a synergistic effect between the two factors; when the SI is less than 1, it indicates an antagonistic effect. The significance level was set at *p* < 0.05.

## 3 Results

### 3.1 General characteristics

A total of 320 participants were included in this study. In this research, the 70th percentile of the occupational fatigue scores was selected as the classification criterion for occupational fatigue. When the score was higher than this percentile, the participant was considered to have occupational fatigue and was coded as 1; when the score was lower than this percentile, the participant was considered to have no occupational fatigue and was coded as 0.

The results showed that there were statistically significant differences in age, gender, marital status, daily working hours, weekly working hours and years of work experience between the occupational fatigue group and the non-occupational fatigue group (*p* < 0.05), as shown in Table 1.

**Table 1:** Analysis of basic characteristics (*n* = 320).

Demographic variables	Group	Sample size	Occupational fatigue (*n* = 98)		Non-occupational fatigue (*n* = 222)		F/t	*P*
			Number of samples	Proportion	Number of samples	Proportion		
Age	<30	40	3	3.06	37	16.67	19.358	0.000
	30–39	173	49	50	124	55.86		
	40–49	76	35	35.71	41	18.47		
	≥50	31	11	11.22	20	9.01		
Gender	Male	73	12	12.24	61	27.48	8.958	0.003
	Female	247	86	87.76	161	72.52		
Marital status	Unmarried	36	3	3.06	33	14.86	10.728	0.005
	Married	277	94	95.92	183	82.43		
	Divorced/Widowed	7	1	1.02	6	2.7		
Daily working hours	<8 h	34	16	16.33	18	8.11	18.847	0.001
	8–9 h	78	34	34.69	44	19.82		
	9–10 h	71	14	14.29	57	25.68		
	10–12 h	88	18	18.37	70	31.53		
	>12 h	49	16	16.33	33	14.86		
Weekly working days	<5d	117	49	50	68	30.63	18.459	0.001
	5–5.5d	118	37	37.76	81	36.49		
	5.5–6d	54	8	8.16	46	20.72		
	6–6.5d	22	2	2.04	20	9.01		
	6.5–7d	9	2	2.04	7	3.15		
Professional title	Primary	209	61	62.24	148	66.67	1.588	0.452
	Intermediate	87	27	27.55	60	27.03		
	Advanced	24	10	10.2	14	6.31		
Years of work experience	2–5 year	115	31	31.63	84	37.84	10.836	0.013
	5–10 year	110	31	31.63	79	35.59		
	10–20 year	42	22	22.45	20	9.01		
	>20 year	53	14	14.29	39	17.57		
Number of children	0	15	6	6.12	9	4.05	3.474	0.324
	1	73	24	24.49	49	22.07		
	2	226	68	69.39	158	71.17		
	≥3	6	0	0	6	2.7		
Education level	Junior college education or lower	26	8	8.16	18	8.11	0.117	0.943
	Bachelor’s degree	264	80	81.63	184	82.88		
	Postgraduate degree and above	30	10	10.2	20	9.01		

### 3.2 Associations between work–family conflict, coping styles and occupational fatigue

Logistic regression analysis was conducted to examine the associations between work–family conflict, positive coping, negative coping, and occupational fatigue. Model 1 did not include moderator variables; Model 2 adjusted for age, gender, marital status, education level and professional title; Model 3 further adjusted for daily working hours, weekly working days, years of work experience and number of children.

The results showed that work–family conflict and negative coping were risk factors for occupational fatigue (*p* < 0.05). In the fully adjusted model (adjusted for age, gender, marital status, education background, professional title, daily working hours, weekly working days, length of service and number of children), the association between high-intensity work–family conflict and occupational fatigue remained significant. The probability of developing occupational fatigue in the high-intensity work–family conflict group was 1.214 times higher than that in the low-intensity work–family conflict group. Moreover, the probability of developing occupational fatigue with negative coping style was 1.209 times higher than that with positive coping style. Detailed results are presented in Table 2.

**Table 2:** Analysis of the association between work–family conflict or coping styles and occupational fatigue.

Variables	Model 1		Model 2		Model 3	
	OR (95%CI)	*P*	OR (95%CI)	*P*	OR (95%CI)	*P*
Work–family conflict	1.206 (1.137–1.278)	0.000	1.212 (1.142–1.286)	0.000	1.214 (1.143–1.289)	0.000
Positive coping	0.829 (0.786–0.874)	0.000	0.811 (0.767–0.858)	0.000	0.800 (0.755–0.848)	0.000
Negative coping	1.185 (1.119–1.255)	0.000	1.203 (1.133–1.278)	0.000	1.209 (1.137–1.285)	0.000

### 3.3 Interaction effects of work–family conflict and coping style on occupational fatigue

#### 3.3.1 Interaction between work–family conflict and negative coping

An additive synergistic interaction was observed between work–family conflict and negative coping in relation to occupational fatigue. After fully adjustment for confounding variables, the relative excess risk due to interaction (RERI) was 0.306 (95% CI: 0.291–0.437), the attributable proportion due to interaction (API) was 0.067 (95% CI: 0.025–0.112), and the synergy index (SI) was 1.295 (95% CI: 1.064–1.489). The synergistic interaction between work–family conflict and negative coping resulted in an occupational fatigue risk that was 1.293 times higher than expected under an additive model. Among them, 6.7% of the risk is attributable to the interaction effect of the two. Detailed statistics are provided in Tables 3, 4.

**Table 3:** Qualitative analysis of the additive interaction between work-family conflict and negative coping on occupational fatigue.

Negative coping	Work–family conflict	Model1		Model 2		Model 3	
		OR (95%CI)	*P*	OR (95%CI)	*P*	OR (95%CI)	*P*
No	No	1.000	–	1.000	–	1.000	–
Yes	No	2.057 (1.145–3.695)	0.016	2.361 (1.268–4.398)	0.007	2.456 (1.284–4.695)	0.007
No	Yes	1.167 (0.849–1.604)	0.340	1.167 (0.834–1.632)	0.367	1.170 (0.834–1.641)	0.363
Yes	Yes	1.366 (1.103–1.692)	0.004	1.442 (1.138–1.826)	0.002	1.473 (1.150–1.886)	0.002

**Table 4:** Quantitative analysis of the additive interaction between work-family conflict and negative coping on occupational fatigue.

Indicators of evaluation	Model 1	Model 2	Model 3
RERI (95%CI)	0.036 (0.021–0.088)	0.368 (0.218–0.441)	0.306 (0.291–0.437)
API (95%CI)	0.010 (0.006–0.055)	0.082 (0.022–0.144)	0.067 (0.025–0.112)
SI (95%CI)	1.111 (1.013–1.612)	1.317 (1.021–1.503)	1.293 (1.064–1.489)

#### 3.3.2 Interaction between work–family conflict and positive coping

An antagonistic interaction was found between work–family conflict and positive coping. After fully adjusting for confounding variables, the relative excess risk due to interaction (RERI) was 0.341 (95% CI: 0.122–0.651), the attributable proportion due to interaction (API) was 0.315 (95% CI: 0.102–0.627), and the synergy index (SI) was 0.700 (95% CI: 0.205–0.990). These findings indicate that when work–family conflict coexists with positive coping, the combined risk of occupational fatigue is approximately 30% lower than the sum of the individual effects. A total of 31.5% of this reduction is attributable to the antagonistic interaction between the two factors. See Tables 5, 6 for detailed results.

**Table 5:** Qualitative analysis of the additive interaction between work–family conflict and positive coping on occupational fatigue.

Positive coping	Work–family conflict	Model1		Model 2		Model 3	
		OR (95%CI)	*P*	OR (95%CI)	*P*	OR (95%CI)	*P*
Yes	No	1.000	–	1.000	–	1.000	–
No	No	1.733 (0.951–3.158)	0.072	2.288 (1.162–4.505)	0.017	2.313 (1.159–4.615)	0.017
Yes	Yes	1.490 (1.091–2.036)	0.012	1.579 (1.136–2.196)	0.007	1.570 (1.115–2.211)	0.010
No	Yes	2.270 (1.702–3.027)	0.000	2.407 (1.768–3.276)	0.000	2.589 (1.863–3.598)	0.000

**Table 6:** Quantitative analysis of the additive interaction between work–family conflict and positive coping on occupational fatigue.

Indicators of evaluation	Model 1	Model 2	Model 3
RERI (95%CI)	0.401 (0.218–0.664)	0.744 (0.217–0.964)	0.341 (0.122–0.651)
API (95%CI)	0.119 (0.021–0.411)	0.198 (0.036–0.503)	0.315 (0.102–0.627)
SI (95%CI)	0.203 (0.023–0.601)	0.371 (0.149–0.830)	0.700 (0.205–0.990)

## 4 Discussion

### 4.1 Impact of work–family conflict on occupational fatigue

This study found that high-intensity work–family conflict significantly increases the risk of occupational fatigue among endoscopy nurses, consistent with previous research findings (9, 30, 31). According to the JD-R Model, work–family conflict is regarded as an important job demand that continuously consumes employees’ psychological and physiological resources, thereby increasing the likelihood of occupational fatigue (32, 33).

Endoscopy nurses often experience highly irregular working hours, and their family routines are frequently disrupted by emergency duties. Physiologically, long-term exposure to work–family conflict can lead to irregular sleep patterns and poor dietary habits, intensifying physical fatigue (34). Psychologically, the combined pressure of demanding work and strained family relationships can induce emotional exhaustion, anxiety, and depression, ultimately reducing nurses’ resilience and motivation (30).

In addition, the high work pressure and special work environment make work–family conflict more prominent. This conflict continuously depletes physical and mental resources, leading to problems such as physical discomfort and low mood (35). In the family domain, the limited time spent with family members may lead to disharmonious family relationships, increasing the psychological burden. The weakening of the social support system restricts the channels for stress relief, further intensifying the sense of occupational fatigue (9, 36). Long-term exposure to high-intensity work–family conflict seriously impacts the occupational identity and work enthusiasm of endoscopy nurses, leading them to doubt their own values (37).

Therefore, reducing work–family conflict is an important strategy for preventing occupational fatigue. Organizations can help employees better balance work and family responsibilities by providing flexible work arrangements (such as flexible working hours and telecommuting) and family support policies (such as parenting support and family—friendly policies), thereby reducing the risk of occupational fatigue.

### 4.2 Effects of coping styles on occupational fatigue

#### 4.2.1 Effects of negative coping on occupational fatigue

The research results indicate that negative coping significantly increases the risk of occupational fatigue among endoscopy nurses. Negative coping styles are typically manifested as behaviors such as withdrawal and denial. Although these strategies may temporarily relieve stress in the short term, in the long run, they significantly weaken an individual’s coping ability and exacerbate the impact of stress on physical and mental health (38, 39).

The research results indicate that negative coping significantly increases the risk of occupational fatigue among endoscopy nurses. Negative coping styles are typically manifested as behaviors such as withdrawal and denial. Although these strategies may provide short-term relief, they tend to weaken an individual’s long-term coping capacity and aggravate the adverse effects of stress on both physical and psychological health (38, 39). Work–family conflict, as a typical job demand, strengthens the use of negative coping styles by increasing an individual’s psychological stress (40). When an individual faces conflicts between work and family, the psychological burden surges, making it easy to fall into a vicious cycle of negative coping.

While our study did not conduct formal mediation analysis, previous research suggests that negative coping may serve as a potential explanatory pathway through which work–family conflict contributes to occupational fatigue (40, 41). Prolonged engagement in negative coping can lead to physiological consequences, such as endocrine imbalances and reduced immune function (42), as well as psychological strain, including anxiety, depression, and reduced motivation (43). In the context of endoscopy nursing, where high job demands and family responsibilities often coincide, nurses who resort to avoidance or withdrawal may find that such strategies exacerbate both the perceived conflict and fatigue symptoms. These findings highlight the importance of addressing coping mechanisms in stress management and occupational fatigue prevention strategies.

Therefore, organizations should take active and effective measures. Firstly, conduct regular training on coping strategies to teach employees positive methods and skills for dealing with stress and improve their coping abilities. Secondly, create a supportive work atmosphere. Managers need to closely monitor the psychological states of employees and provide them with timely psychological support and guidance. Finally, organizations should offer professional mental health services to help employees relieve psychological stress and break the vicious cycle between negative coping and occupational fatigue.

#### 4.2.2 Effects of positive coping on occupational fatigue

This study indicates that positive coping styles significantly buffer the adverse effects of work–family conflict on occupational fatigue of work–family conflict on the occupational fatigue of endoscopy nurses. According to the JDR Model, positive coping styles can be regarded as a kind of work resource, which can help endoscopy nurses effectively manage stress and reduce the occurrence of occupational fatigue (9).

Positive coping works through resource conservation and emotional regulation mechanisms, enhancing psychological resilience and helping individuals maintain equilibrium when faced with complex work–life demands (44). A constructive mindset boosts confidence in problem-solving and reduces stress from time-related conflicts (45). In addition, positive coping helps reduce the occurrence of anxiety, depression, and emotional exhaustion, thereby supporting mental health and reducing occupational fatigue. Previous studies have shown that nurses who adopt positive coping strategies experience significantly lower levels of occupational fatigue than those who rely on negative coping (46).

Therefore, cultivating and enhancing the positive coping abilities of endoscopy nurses is an important means of preventing occupational fatigue. Organizations can provide training on time management, problem—solving, and social skills to help endoscopy nurses learn positive coping styles, improve their psychological resilience, and enhance their ability to cope with stress. In addition, organizations can also create a supportive work atmosphere, encourage endoscopy nurses to seek help and share experiences, and further strengthen the effect of positive coping.

### 4.3 Interaction effects

#### 4.3.1 Synergistic effect of negative coping

Due to the nature of their profession, endoscopy nurses often face complex and high—pressure work environments, frequently dealing with emergencies and high—intensity tasks. Work–family conflict is particularly prominent in this context. It intensifies psychological stress and reinforces the use of negative coping styles (such as withdrawal and denial) (47). When endoscopy nurses remain in a prolonged state of work–family conflict, their psychological resources (including emotion regulation ability and problem-solving ability) are gradually depleted. The use of negative coping styles further weakens their ability to cope with stress, creating a vicious cycle that significantly exacerbates occupational fatigue.

#### 4.3.2 Antagonistic effect of positive coping

In contrast to negative coping, positive coping strategies appear to buffer the adverse effects of work–family conflict on fatigue through mechanisms of resource conservation and emotional regulation. For instance, effective time management enables nurses to better balance professional and family responsibilities, reducing stress caused by role conflict (9).

Moreover, positive coping strengthens psychological resilience, helping nurses maintain emotional stability and physical health under pressure (48). The emotion regulation mechanism helps endoscopy nurses maintain mental health by reducing the occurrence of negative emotions such as anxiety and depression, further alleviating the degree of occupational fatigue (49). Positive coping styles such as problem-solving and social support can effectively relieve the psychological stress brought about by work–family conflict, thereby reducing the risk of occupational fatigue.

Based on the JD-R model, this study concludes that work–family conflict and negative coping produce a synergistic effect via resource depletion, significantly increasing the risk of occupational fatigue. In contrast, positive coping exerts an antagonistic effect by preserving resources and regulating emotions, thereby offsetting part of the adverse impact. Therefore, reducing work–family conflict, suppressing negative coping strategies, and strengthening positive coping abilities are the key approaches to preventing occupational fatigue among endoscopy nurses.

## 5 Limitations of the study and future research directions

### 5.1 Limitations

(1) This study adopted a cross-sectional design, which restricts the ability to establish causal relationships. Future studies should consider using longitudinal designs to examine the dynamic interactions among work–family conflict, coping styles, and occupational fatigue over time.
(2) The evaluation of occupational fatigue relied solely on self-reported scales, which may be influenced by subjective biases such as social desirability or recall error. To enhance the objectivity and accuracy of fatigue assessment, future research could incorporate physiological indicators (such as sleep quality, heart rate variability) and behavioral data.
(3) Although participants were drawn from 23 medical institutions across 14 provincial-level regions in China, the total sample size of 320 and the focus on a single occupational group (endoscopy nurses) may limit the external generalizability of the findings. Additionally, regional differences in institutional resources, workload intensity, and support systems may have influenced participants’ experiences and responses.
(4) The study was conducted exclusively within the Chinese healthcare system, which may differ from other countries in terms of nurse–patient ratios, organizational support, cultural norms, and expectations regarding family responsibilities. These contextual differences may influence the manifestation of work–family conflict, coping behaviors, and perceptions of fatigue. Future research should aim to replicate or validate these findings in different cultural and healthcare settings to enhance cross-national applicability.

### 5.2 Future research directions

(1) To address the limitations of cross-sectional data, future studies should employ longitudinal designs that capture the evolving and causal relationships among work–family conflict, coping styles, and occupational fatigue. This approach can reveal how these variables interact over time and under changing work-life conditions.
(2) Future research should supplement self-reported scales with objective, multidimensional assessment tools. This may include the use of physiological measures, behavioral tracking or digital biomarkers to enhance the accuracy and reliability of occupational fatigue assessments.
(3) To enhance generalizability, subsequent studies should recruit larger and more diverse samples, including nurses from various specialties and healthcare settings. Stratified or multi-stage sampling across different geographic and institutional contexts can help account for regional disparities in workload, resource allocation, and institutional support.
(4) Given the influence of cultural norms, healthcare structures, and family-role expectations, future research should replicate or validate the current findings in diverse cultural and national contexts. In addition, Intervention studies can be carried out to evaluate the effectiveness of different intervention measures in reducing occupational fatigue, providing a scientific basis for practice.

## 6 Conclusion

From the perspective of the JD-R Model, this study explored the relationships among work–family conflict, coping styles, and occupational fatigue. The research results indicate that work–family conflict and negative coping are significant risk factors for occupational fatigue, while positive coping can, to a certain extent, buffer the negative impacts brought about by work–family conflict. This finding provides important theoretical basis and practical guidance for organizational management practices, emphasizing the significance of comprehensively considering work–family conflict and coping styles in occupational health management. By reducing work–family conflict, enhancing work resources, and cultivating positive coping styles, organizations and employees can effectively prevent occupational fatigue and improve employees’ physical and mental health as well as work performance. Future research can further explore the long—term impacts among work–family conflict, coping styles, and occupational fatigue, as well as the moderating effects of factors such as culture and social support.

## Data Availability

The original contributions presented in the study are included in the article/supplementary material, further inquiries can be directed to the corresponding author.
