# Correction: The interactive effects of work–family conflict and coping styles on occupational fatigue among endoscopy nurses: a cross-sectional study

**DOI:** 10.3389/fpubh.2025.1682758

**Published:** 2025-08-29

**Authors:** Zhi Zeng, Chunyan Fu, Sumei Zhou, Guiqiong Xie, Yazhi He, Meng Liu, Chao Liu

**Affiliations:** ^1^Department of Gastroenterology, Deyang People's Hospital, Deyang, Sichuan, China; ^2^Pediatric Ward 2 (Children's Blood/Cancer Ward), Sichuan Provincial People's Hospital, Chengdu, China; ^3^Department of Neurosurgery, Deyang People's Hospital, Deyang, Sichuan, China

**Keywords:** endoscopy nurses, occupational fatigue, work–family conflict, coping style, JD-R model, interactive effects

The ethical approval details were erroneously given as No. 2023-05-042-K01, it should be No. 2023-04-083-K01.

This has been corrected in section **2 Materials and methods**, *2.1 Participants*, paragraph 5: “This study was reviewed and approved by the Ethics Committee of Deyang people's Hospital (No. 2023-04-083-K01). All participants provided informed consent and voluntarily participated in the study.”

The corrected **Ethics statement** appears below:

“The studies involving humans were approved by Ethics Committee of Deyang People's Hospital (No. 2023-04-083-K01). The studies were conducted in accordance with the local legislation and institutional requirements. The participants provided their written informed consent to participate in this study.”

The original version of this article has been updated.

